# Plasma interleukin-23 and circulating IL-17A^+^IFNγ^+^ ex-Th17 cells predict opposing outcomes of anti-TNF therapy in rheumatoid arthritis

**DOI:** 10.1186/s13075-022-02748-3

**Published:** 2022-02-26

**Authors:** Melanie J. Millier, Niamh C. Fanning, Christopher Frampton, Lisa K. Stamp, Paul A. Hessian

**Affiliations:** 1 Department of Medicine, University of Otago, P.O. Box 56, Dunedin, 9054 New Zealand; 2 Department of Medicine, University of Otago, Christchurch, P.O. Box 4345, Christchurch, 8014 New Zealand

## Abstract

**Objectives:**

TNF-α inhibitors are widely used in rheumatoid arthritis (RA) with varying success. Response to TNF-α inhibition may reflect the evolution of rheumatoid inflammation through fluctuating stages of TNF-α dependence. Our aim was to assess plasma concentrations of Th-17-related cytokines and the presence of circulating effector T-cells to identify predictors of response to TNF-α inhibitors.

**Methods:**

Ninety-three people with RA were seen prior to and 4–6 months after commencing etanercept or adalimumab. Plasma concentrations of Th17-related cytokines, circulating effector T-cells, their production of relevant transcription factors and intracellular cytokines were measured at baseline. EULAR response criteria were used to define poor (ΔDAS28 ≤ 1.2 and/or DAS28 > 3.2) and good (ΔDAS28 > 1.2 and DAS28 ≤ 3.2) responders. Multivariate logistic regression was used to identify predictors of response.

**Results:**

Participants with plasma IL-23 present at baseline were more likely to be poor responders [15/20 (75%) of IL-23^+^ versus 36/73 (49.3%) of IL-23^−^; *p* = 0.041]. While frequencies of Th1, Th17, ex-Th17 and T_reg_ cell populations were similar between good and poor responders to anti-TNF therapy, IL-17A^+^IFNγ^+^ ex-Th17 cells were more prevalent in good responders (0.83% of ex-T_H_17 cells) compared to poor responders (0.24% of ex-Th17 cells), *p* = 0.023. Both plasma IL-23 cytokine status (*OR* = 0.17 (95% *CI* 0.04–0.73)) and IL-17A^+^IFNγ^+^ ex-Th17 cell frequency (*OR* = 1.64 (95% *CI* 1.06 to 2.54)) were independently associated with a good response to anti-TNF therapy. Receiver operator characteristic (ROC) analysis, including both parameters, demonstrated an area under the ROC curve (AUC) of 0.70 (95% *CI* 0.60–0.82; *p* = 0.001).

**Conclusions:**

Plasma IL-23 and circulating IL-17A^+^IFNγ^+^ ex-Th17 cells are independently associated with response to anti-TNF therapy. In combination, plasma IL-23 and circulating IL-17A^+^IFNγ^+^ ex-Th17 cells provide additive value to the prediction of response to anti-TNF therapy in RA.

**Supplementary Information:**

The online version contains supplementary material available at 10.1186/s13075-022-02748-3.

## Introduction

Rheumatoid arthritis (RA) is a chronic autoimmune disease characterised by synovial joint inflammation that, without adequate treatment, leads to destruction of joint cartilage and bone. RA pathology is heterogeneous, arising from a spectrum of genetic, molecular and cellular mechanisms that varyingly control the presentation and perpetuation of the disease. Consequently, treatment and its efficacy are similarly heterogeneous and unpredictable [1, 2]. Sub-types of RA inflammation may be distinguished by histologic features and signature expression profiles of synovial tissue — knowledge which may hold promise for guiding treatment options [2–6].

The advent of biological disease-modifying anti-rheumatic drugs (bDMARDs), including anti-tumour necrosis factor (TNF) therapy, has revolutionised the treatment of RA and made remission a realistic goal for many people with RA. TNF-α is a pivotal pro-inflammatory cytokine in RA that stimulates a variety of cell types in the joint space to promote inflammation and cartilage degradation [7, 8], while also contributing to disease chronicity [9]. The importance of TNF-α signalling in driving RA pathogenesis is highlighted by the potential for anti-TNF therapies to arrest radiographic damage [10]. However, approximately 30–40% of people with RA do not respond to anti-TNF therapy when used as the first-line bDMARD [1, 7].

Evidence from RA synovial tissue analysis suggests myeloid-dominant inflammation may be more amenable to anti-TNF therapy than other classes of biologic therapies, such as those targeting interleukin (IL)-6 or CD20 [4, 11]. Furthermore, recent evidence implicates dysregulation of the T-helper (Th)17 cell plasticity within the inflamed joint as an important contributor to the onset, chronicity and progression of RA [12–14]. TNF-α has been shown to co-mediate both the differentiation of Th17 cells [15] and their progression to the more pathogenic, pro-inflammatory ex-Th17 (also known as Th17.1/non-classic Th1) phenotype, capable themselves of producing much higher amounts of TNF-α than their Th17 precursor [16, 17]. Some studies have reported circulating Th17 cells and high pre-treatment levels of their prototypic cytokine, IL-17A, are associated with poor response to anti-TNF therapy [18–20]. However, there is a collective lack of consensus between various studies investigating the predictive value of circulating biomarkers, either protein or cellular, in RA therapy [21].

The high cost of anti-TNF therapies, the possibility of further disease progression in non-responders and the potential risk of adverse reactions to therapy necessitate an approach to more accurately predict the outcome of anti-TNF therapy and dictate the most effective course of treatment. Hence, our objective was to explore whether screening plasma levels of Th17-related cytokines and peripheral T-cell populations in RA patients immediately prior to commencing anti-TNF therapy could identify a blood-based biomarker profile that associates specifically with clinical improvement in disease activity after the initial phase of treatment.

## Materials and methods

### Participants

Ninety-three biologic-naïve people with RA as defined by the American College of Rheumatology (ACR) classification criteria [22], commencing anti-TNF therapy (etanercept *n* = 20 or adalimumab *n* = 73), were recruited from three public hospitals. The study was approved by the University of Otago Ethics Human Health Committee (13/040). All participants provided written informed consent. Choice of therapy was at the discretion of the treating clinician in conjunction with patient preference. The criteria for anti-TNF therapy in New Zealand require people to have had RA for at least 6 months; to have radiographic evidence of erosions or be anti-cyclic citrullinated peptide antibody (ACPA) positive; to have failed therapy with (i) methotrexate alone, (ii) methotrexate in combination with salazopyrin and hydroxychloroquine, and (iii) leflunomide or cyclosporine; to have ≥4 large or 20 total active joints; and to have a C-reactive protein (CRP) > 15mg/L unless they have received concomitant prednisone at doses > 5mg per day for the previous 3 months.

Participants were assessed immediately prior to commencing anti-TNF therapy (baseline) and 4 to 6 months after commencing anti-TNF therapy (follow-up). Demographic details and clinical history were recorded by participant questionnaire and review of the medical record. Disease activity was assessed at each visit using standard clinical parameters: 28 tender and 28 swollen joint counts, CRP and patient’s global assessment of disease activity. DAS28-4V-CRP disease activity score (DAS28) was calculated at each visit and ∆DAS28 was calculated as the difference between baseline and follow-up DAS28 scores. Blood samples were obtained at both baseline and follow-up visits and processed for later inclusion in cytokine assays or multiplex flow cytometry analysis.

### Determining response to treatment

Good response to treatment with anti-TNF therapy was defined by European League Against Rheumatism (EULAR) response criteria: low disease activity at the follow-up visit (DAS28 ≤3.2) and a change in DAS28 score greater than 1.2 (∆DAS28 >1.2) between baseline and follow-up visits [23]. Participants with follow-up DAS28 >3.2 and/or ∆DAS28 ≤1.2 were classified as poor responders to therapy.

### Circulating cytokine measurement

Plasma concentrations of Th17-related cytokines were determined using the 15-plex magnetic bead-based Bio-Plex Pro™ Human Th17 Cytokine Panel kits (171AA001M) run on a calibrated Bioplex 200 System (Bio-Rad Laboratories, Inc.) and analysed using Milliplex Analyst 5.1 analysis software (VigeneTech, Inc.) as previously described [24].

Cytokines detected in ≤ 50% of samples at baseline were dichotomised (IL-4, IL-17A, IL-17F, IL-21, IL-22, IL-23, IL-25, interferon (IFN)-γ and sCD40L), and the remaining cytokines (IL-1β, IL-6, IL-10, TNF-α, IL-31 and IL-33), detected in > 50% of participants, were treated as continuous variables. A measure above the lower limit of quantitation (LLOQ) was considered positive and samples below the LLOQ for each individual assay were assigned a value of zero.

### Flow cytometry

Peripheral blood mononuclear cells (PBMC) were immediately isolated from EDTA-treated blood samples using density gradient centrifugation with Ficoll-Paque Plus (GE Healthcare). To facilitate batch processing, the isolated PBMC were stored in liquid nitrogen at 1 × 10^7^ cells/mL in 90% foetal calf serum (FCS)/10% di-methyl sulphoxide solution, following a controlled rate freezing step. Upon thawing at 37 °C, cells were washed in PBS/2%FCS then rested overnight in a 37°C, 5% CO_2_ humidity chamber at 1 × 10^6^ viable cells/mL in 10% FCS (Gibco), 1% penicillin-streptomycin (Sigma-Aldrich)-supplemented RPMI 1640 media (Gibco). Eighty-four of 93 samples contained sufficiently viable cells (> 80%) for inclusion in the PBMC analysis.

Each sample was divided in two, and each portion incubated in a cocktail of Brilliant Stain Buffer (Becton Dickinson [BD] Horizon), Fc Block (BD Pharmingen), PBS/2%FCS, and fluorescent-labelled antibodies specific for detection of cell surface markers for either Th cells or T_reg_ cells. Cells were stimulated with 125 ng/mL media phorbol 12-myristate 13-acetate (PMA) and 2.5 ug/mL Ionomycin using leukocyte activation cocktail with BD GolgiPlug (BD Pharmingen) and PMA (Sigma-Aldrich) for 6 h at 37°C in a 5% CO_2_ humidity chamber. Cells were incubated with Fixable Viability Stain (FVS585V, BD Horizon), prior to fixation and permeabilisation using Transcription Factor Buffer Set (BD Horizon) and parallel incubation with separate post-permeabilisation antibody cocktails that distinguish either Th-effector cell types or T_reg_ cells.

Stained and washed cells were resuspended and analysed on a BD LSRFortessa flow cytometer with FACSDiva software (BD Bioscience). An average of 42,817 events were acquired for analysis within the singlet/live/lymphocyte/CD3^+^/CD4^+^ gate and software application settings were used to maintain consistent placement of the negative populations between multiple runs.

### Immunophenotyping

Effector T-cell populations were identified from within the circulating CD3^+^/CD4^+^ lymphocyte population, derived from single, live events, using FlowJo software (version 10.6.1, BD). Fluorescence-labelled antibody panels (detailed in Additional file 1) were used to stain and identify Th17 cells: CXCR3^−^/CD161^+^/CCR4^+^/CCR6^+^; ex-Th17 cells: CXCR3^+^/CD161^+^/CCR6^+^/CCR4^+/−^; and Th1 cells: CXCR3^+^/CD161^−^/CCR4^−^/CCR6^−^. T_reg_ cells were identified in a parallel sample as a distinct sub-population of CD3^+^/CD4^+^/CD25^+^/CD127^lo^ lymphocytes (see Additional file 2 for gating strategies). Additionally, podoplanin (PDPN) expression, intracellular cytokines IL-17A and IFNγ, and RORγT transcription factor expression were assessed within Th17, ex-Th17 and Th1 cells, while intracellular IL-17A and both RORγT and FOXP3 transcription factor expression were assessed within T_reg_ cells (Additional file 1).

Gating thresholds were determined for both surface markers and intracellular cytokines by comparison with respective fluorescence minus one (FMO) controls (Additional file 2). For FMO controls, positive gates contained 0–0.1% cells. Gating thresholds for intracellular cytokines were further validated by comparisons with unstimulated, stained, control samples. In these unstimulated control samples, a varying percentage of positive cells were detected, reflecting pre-existing intracellular cytokine production. Further validation of IL-17A staining specificity was enabled by determining co-expression of RORγT in stimulated samples (Additional file 2). All FACS data reported is from stimulated and stained cells.

### Statistical analysis

Based on indices of EULAR response criteria at follow-up, all participants were categorised as either good or poor responders. Between the two response groups, comparisons of participants’ original baseline demographics or clinical variables were performed using Pearson’s chi-square tests, Student’s *T* tests or Mann-Whitney *U* tests where appropriate. Statistical significance was defined as a two-tailed *p* value < 0.05.

For each dichotomously treated cytokine, good or poor response rate was compared between cytokine-present or cytokine-absent groups, using Pearson’s chi-square or Fisher’s exact test. The baseline concentration of cytokines with continuous data treatment was compared between participants showing good or poor response using the Mann-Whitney *U* test.

For flow cytometry analysis, the median of the frequency of various cell populations or the median fluorescence intensity (MFI) for marker expression was compared between participant groups showing good or poor response using Mann-Whitney *U* tests.

Univariable and multivariable binary logistic regression, using good response to anti-TNF treatment as the outcome, was performed with quartile-transformed double-positive IL-17A^+^IFNγ^+^ ex-Th17 cell frequencies and IL-23 as independent variables. To determine the combined effects of IL-23 status and IL-17A^+^IFNγ^+^ ex-Th17 cell frequency on the likelihood of a good response, the predicted probabilities from the multivariate logistic regression were used in the receiver operating characteristic curve (ROC) curve analysis.

### Data statement

The datasets generated and/or analysed during the current study are the subject of ongoing analysis and will be available from the corresponding author on reasonable request and subject to ethical approval.

## Results

### Participant demographics

The baseline demographics and clinical variables of the two response groups are summarised in Table 1. Two-thirds of the participants had disease duration less than 10 years, and the mean baseline DAS28 score was 5.07 (*SD* 1.23), with an overall moderate to high disease activity reflected in a DAS28 score above 3.2 in 92.5% of patients. At the follow-up visit, 45% of participants were deemed good responders, while 55% were poor responders according to established EULAR criteria. Individual baseline demographic variables, disease characteristics or existing pharmaceutical treatment regime were not different between good and poor responders (Table 1). Similarly, within the sub-cohort of 84 participants included in the PBMC analysis, demographic variables, disease characteristics or treatments were not different between good and poor responders (data not shown), and all variables within this group were representative of the overall cohort.

**Table 1:** Biologic-naïve RA patient demographics and clinical variables at baseline

Baseline measures	EULAR response outcome		***P*** value^**a**^
	Good	Poor	
Number	42	51	
Female, *n* (%)	29 (69)	34 (67)	*0.81*
Age, mean years ± SD	56.5 ± 12.8	58 ± 13.7	*0.58*
DAS28, mean ± SD	5.08 ± 1.08	5.07 ± 1.36	*0.97*
DAS28 >3.2, *n* (%)	40 (95)	46 (90)	*0.36*
CRP, mg/L median (IQR)	11.5 (3–21)	11 (3–28)	*0.86*
RF+, *n* (%)	36 (86)	39 (77)	*0.26*
ACPA+^b^, *n* (%)	32 (80)	41 (80)	*0.96*
Nodules present^c^, *n* (%)	16 (39)	18 (35)	*0.71*
Erosions present, *n* (%)	39 (93)	46 (90)	*0.65*
Years since diagnosis, median (IQR)	7.6 (3.2–18.0)	6.3 (2.6–13.4)	*0.37*
**TNF inhibitor commenced**^**d**^			
Adalimumab, *n* (%)	31 (74)	42 (82)	*0.32*
Etanercept, *n* (%)	11 (26)	9 (18)	*0.32*
**Concomitant DMARDS**			
Prednisone, *n* (%)	30 (71)	41 (80)	*0.31*
Prednisone dose^e^, mg, median (IQR)	10 (6.0–15.0)	10 (6.0–15.0)	*0.89*
Methotrexate, *n* (%)	25 (60)	24 (47)	*0.23*
Leflunomide, *n* (%)	11 (26)	11 (21)	*0.89*
Salazopyrin, *n* (%)	2 (5)	5 (10)	*0.36*
Hydroxychloroquine (%)	9 (21)	6 (12)	*0.21*

^a^Pearson’s chi-square test was used to compare frequency. Student’s *T* test was used to compare mean. The Mann-Whitney *U* test was used for comparing median

^b^Two participants had no record of ACPA measurement due to the test not being available at the time of diagnosis

^c^One participant did not have the presence/absence of nodules confirmed

^d^Low numbers within a divided cohort precluded any reliable comparison between adalimumab and etanercept treated participants

^e^Median dose in prednisone-only participants. Information on prednisone dose was not available for one participant

### The presence of plasma IL-23 at baseline is associated with poor response to anti-TNF therapy

**Fig. 1:**
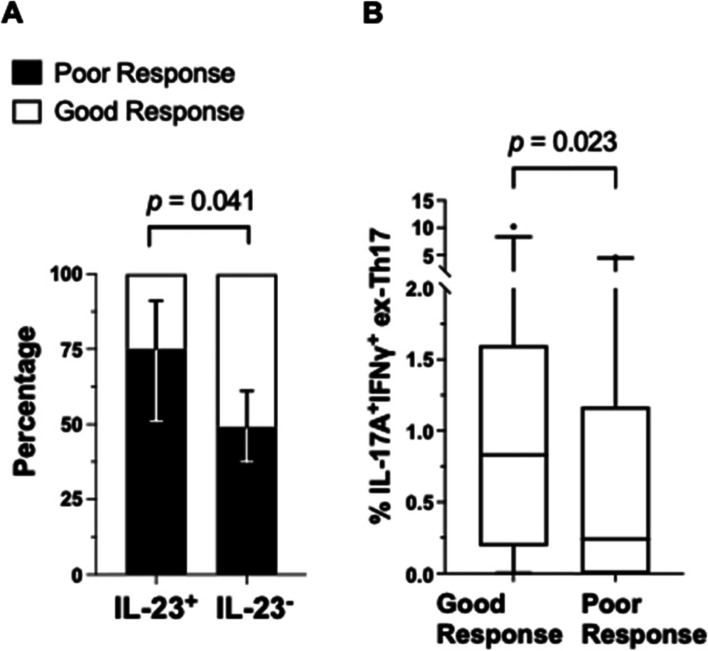
Baseline blood profiles associated with response to anti-TNF therapy. **A** Presence of plasma IL-23 (IL-23^+^) at baseline and response to anti-TNF therapy. Depicted is the frequency of poor response with 95% confidence interval error bars in parenthesis for only IL-23^+^ participants (*n*=20): 75% (51–91%); and for only IL-23^−^ participants (*n*=73): 49% (37–61%). A significantly higher proportion of IL-23^+^ participants at baseline are poor responders (*p* = 0.041). **B** Circulating IL-17A^+^IFNγ^+^ ex-Th17 cells at baseline and response to anti-TNF therapy. The frequency of IL-17A^+^IFNγ^+^ ex-Th17 cells is significantly higher in good responders (*p*=0.023). Data are expressed as the median and interquartile range with 5% and 95% percentiles indicated

Of the 15 select Th17-related cytokines measured (detailed in Additional file 3), a statistically significant association with response to anti-TNF therapy was only seen for plasma IL-23 (*p*=0.041). Of only those participants where IL-23 cytokine was present at baseline (IL-23^+^), 75% were poor responders to anti-TNF therapy, compared to only participants lacking IL-23 at baseline (IL-23^−^), where 49% were poor responders (Fig. 1A).

### Higher frequencies of IL-17A^+^IFNγ^+^ exT_H_17 cells are associated with good response to anti-TNF therapy

PBMC isolated from baseline blood samples were subjected to multiplex flow cytometric analysis, where an immunophenotyping strategy based on specific cell surface marker expression was employed to identify and quantitate cell sub-populations (Additional file 4). Comparisons were made between good and poor responders. As summarised in Table 2, interrogation of CD3^+^CD4^+^ T-lymphocytes showed similar frequencies and ratios of surface-marker-defined Th1, Th17, ex-Th17 and T_reg_ cell populations between good and poor responders to anti-TNF therapy.

**Table 2:** Median PBMC cell frequencies at baseline in RA patients subsequently classed as either good or poor responders to anti-TNF therapy

	Good responders (*n*=39)	Poor responders (*n*=45)	*P* value
***Lymphocytes***			
CD3^+^CD4^+^	53.5^a^ (44.8–61.6)	52.6 (43.7–61.1)	0.968
***CD3***^***+***^***CD4***^***+***^			
^a^Th1	8.36 (4.87–13.00)	9.34 (5.83–13.70)	0.667
T_reg_	4.49 (3.70–5.44)	4.43 (3.42–5.22)	0.647
Th17	1.32 (0.73–1.94)	1.37 (0.54–2.09)	0.904
ex-Th17	0.64 (0.32–1.16)	0.48 (0.22–1.26)	0.451
***ex-Th17***			
^a^IFNγ^+^	8.73 (6.06–12.20)	11.0 (7.14–14.90)	0.131
IL-17A^+^IFNγ^+^	0.83 (0.19–1.60)	0.24 (0.00–1.16)	0.023*
IL-17A^−^IFNγ^+^	7.08 (5.15–10.41)	9.18 (6.23–14.28)	0.115
IL-17A^+^	2.39 (0.95–3.93)	1.82 (0.94–4.51)	0.771
***Cell ratios***			
Th1:Th17	6.0 (4.4–12.8)	7.3 (3.7–18.9)	0.69
Th1:ex-Th17	12.1 (7.3–24.5)	16.9 (9.1–32.1)	0.422
Th17:ex-Th17	1.8 (1.4–4.7)	1.9 (1.1–5.2)	0.854
T_reg_:Th17	3.7 (2.5–5.3)	4.1 (2.1–6.0)	0.897

^a^Frequency values for indicated PBMC cellular sub-types are median percentages with IQR in parenthesis of the indicated parent cell populations (shown in bold). Comparisons of each cellular sub-type between patients demonstrating a good or poor response to anti-TNF therapy are shown (Mann-Whitney *U* tests) with *p<0.05 considered statistically significant

Analysis within T-effector cell populations, focusing on IL-17A and IFNγ production, further characterised the various sub-populations (Table 2 and Additional file 4). Within the ex-TH17 cell population, a higher median frequency of cells co-producing IL-17A and IFNγ (IL-17A^+^IFNγ^+^ ex-Th17 cells) were seen in good responders (0.83% of ex-T_H_17 cells) compared to poor responders (0.24% of ex-Th17 cells), *p* = 0.023 (Table 2 and Fig. 1B). The various remaining cell sub-populations showed no statistically significant associations with response to anti-TNF therapy (Additional file 4).

### The combination of pre-treatment plasma IL-23 cytokine status and IL-17A^+^IFNγ^+^ ex-Th17 cell frequency predicts response to anti-TNF therapy

**Fig. 2:**
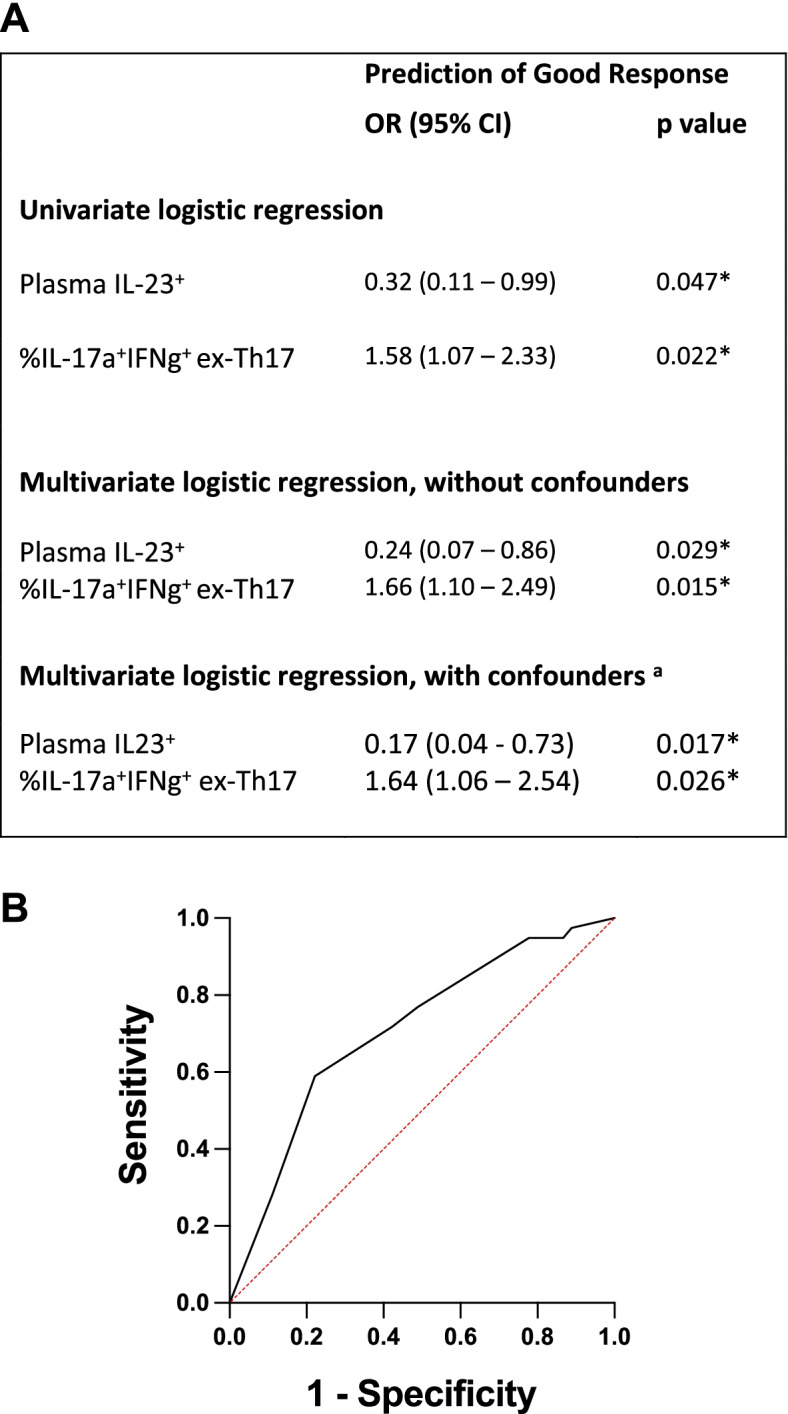
Predicting good response to anti-TNF therapy: independent and combined strength of baseline plasma IL-23^+^ status and frequency of circulating IL-17A^+^IFNγ^+^ ex-Th17 cell sub-population. **A** Uni- and multivariate logistic regression models with baseline variables for the prediction of good response after an initial 4–6 months of anti-TNF treatment. Values for IL-17A^+^IFNγ^+^ ex-Th17 cell frequency were quartile-transformed; plasma IL-23^+^ defined as cytokine present above detectable levels. *Significant *p* values ≤ 0.05. **B** ROC curve analysis to determine the combined effects of IL-23 status and IL-17A^+^IFNγ^+^ ex-Th17 cell frequency on the likelihood of a good response to anti-TNF therapy. Predicted probabilities from the multivariate logistic regression analysis were used. ^a^Odds ratios of this model are adjusted for additional confounders including concomitant cDMARD use (any of methotrexate, leflunomide, salazopyrin and/or hydroxychloroquine); presence of subcutaneous rheumatoid nodules; RF^+^ and/or ACPA^+^; prednisone use; disease duration; and age at baseline

Having established that baseline presence of IL-23 in plasma and a higher frequency of IL-17A^+^IFNγ^+^ ex-Th17 cells associate with divergent response to anti-TNF (Fig. 1 and Table 2), we next explored the combination of these two variables in the prediction of good response to anti-TNF therapy. Multivariable logistic regression analysis, including both IL-17A^+^IFNγ^+^ ex-Th17 cell frequency and plasma IL-23 cytokine status as co-variates, showed each measure was independently associated with a good response to anti-TNF therapy (Fig. 2A).

Any potential influence from individual clinical characteristics (presence of subcutaneous rheumatoid nodules; RF^+^ and/or ACPA^+^; disease duration; age at baseline) and/or concomitant therapies (prednisone use; any of methotrexate, leflunomide, salazopyrin and/or hydroxychloroquine) was considered in an expanded multivariable logistic regression model. This model maintained the independent association of both frequency of IL-17A^+^IFNγ^+^ ex-Th17 cells [*OR* = 1.64 (95% *CI* 1.06 to 2.54)] and IL-23^+^ status [*OR* = 0.17 (95% *CI* 0.04–0.73)] with a good response to anti-TNF therapy (Fig. 2A). Furthermore, the predicted probabilities from multivariate logistic regression that included both parameters demonstrated an area under the ROC curve (AUC) of 0.70 (95% *CI* 0.60–0.82; *p* = 0.001), highlighting the value in combining both IL-23 status and IL-17A^+^IFNγ^+^ cell frequency in the prediction of good response to anti-TNF therapy (Fig. 2B).

## Discussion

This study identified potential biomarkers for the prediction of clinical response to anti-TNF therapy among Th17-related plasma cytokines and circulating effector T-cells in a biologic-naïve RA cohort. Among cytokines, the presence of IL-23 in pre-treatment blood predicted a poor response to anti-TNF therapy. In addition, within baseline PBMC samples, immunophenotyping analysis revealed a small T-cell population, defined by surface marker expression profile as ex-Th17 cells (also recognised as non-classic Th1, or Th17.1 cells), in which both IL-17A and IFNγ were co-expressed. Higher frequencies of this IL-17A^+^IFNγ^+^ ex-Th17 cell population at baseline predicted a good response to anti-TNF therapy. Incorporating both baseline plasma IL-23^+^ cytokine status and IL-17A^+^IFNγ^+^ ex-Th17 cell frequency increased the strength of the response prediction model. These current findings provide insight into inflammatory mechanisms in RA, known to be co-mediated by TNF-α, and with potential to influence response to anti-TNF therapy.

Both the source and functional impact of TNF-α require consideration in order to reconcile the involvement of IL-17A^+^IFNγ^+^ ex-Th17 cells and IL-23 in response to anti-TNF therapy. In accord with published studies [25, 26], baseline plasma TNF-α levels within our RA cohort gave no indication of response to anti-TNF therapy. This suggests a more subtle impact from TNF-α, incorporating potential variations that are contingent upon the balance between inflammatory processes that establish or maintain RA at any juncture, through early- or late-stage disease. Among alternative possibilities, TNF-α makes a contribution towards the differentiation of IL-17A producing Th17 cells [15] and subsequent synergy with IL-17A further increases the potency of TNF-α [8]. Pathogenic Th17 cells are a feature of RA, distinguished by a pro-inflammatory gene signature that includes elevated TNF transcript in non-responders [27] and is sustained by *USF2* signalling pathways [28]. Importantly, TNF-α production by Th17 lineage cells increases as the cells trans-differentiate from classic Th17 cells, through IL-17A^+^IFNγ^+^ ex-Th17 intermediaries, towards non-classical IFNγ^+^ Th1 cell phenotypes, which concomitantly acquire a more pathogenic phenotype [29]. Th17 trans-differentiation appears inherently biased in RA [18, 19] with evidence that the process is TNF-α dependent [17]. Consequently, it has been proposed that TNF inhibitors reduce the shift of Th17 cells towards the ex-Th17/non-classic Th1 cells [30]. A recent report revealing anti-TNF-induced alterations in the blood transcriptome, including reduced expression of the *CD39*/*ENTPD1* gene [31], which is highly expressed in IL-17A^+^IFNγ^+^ ex-Th17 cells, supports this speculation, as does our finding that IL-17A^+^IFNγ^+^ ex-Th17 cells are elevated in the peripheral blood of participants who respond to anti-TNF therapy. Finally, although the derivation of the IL-17A^+^IFNγ^+^ ex-Th17 cells during Th17 trans-differentiation provides a fitting explanation, recent evidence demonstrates increased plasticity of Th17.1/non-classic IFNγ^+^ Th1 cells [32] fostering the opposite conversion towards the Th17 phenotype, including IL-17A^+^IFNγ^+^ ex-Th17 cells. Our data do not allow discrimination of the exact route involved.

We found that the presence of IL-23 in baseline plasma predicted poor response to anti-TNF therapy. Among IL-23^+^ participants that responded poorly, there was no evidence that at baseline the presence of IL-23 per se or measured plasma levels were associated with increased disease activity, nor the extent or features of the effector cells considered (data not shown). Subsequently, IL-23 status was variably affected by anti-TNF therapy, suggesting the in vivo pathogenic mechanisms that involve IL-23 and reduce clinical efficacy are mainly independent of TNF-α. One implication is that IL-23 contributes towards alternative, immune-driven process(es), as shown by the importance of monocyte-derived IL-23 for synovial IL-17A expression [33] and the necessity for IL-23 in development of the pathogenic Th17 cells seen in RA [27]. While it is clear that most of the actions of IL-23 are mediated through IL-17, the disparate predictions for anti-TNF therapy outcome from the presence of IL-23 compared to IL-17A^+^IFNγ^+^ ex-Th17 cells indicate a potential disconnect in the IL-23/IL-17 axis. The presence of IL-23 might “simply” reflect the outcome of a pathological process, as exemplified by the IL-1β-dependent induction of IL-23 p19 in fibroblast-like synoviocytes [34]. More likely, IL-23 is involved in multiple, independent but parallel processes as indicated by activation of the IL-23 pathway, which is strongly associated with synovial ectopic lymphoid neogenesis (ELN) in human RA, yet is seemingly independent of the IL-17A expression necessary for ELN development [6, 35]. Conceivably, the situation might also reflect a temporal effect from IL-23, consistent with evidence suggesting the IL-23/Th17 cell-cytokine(s) axis may not have a constant influence throughout the disease course in RA [36–38]. That IL-23 and IL-17A^+^IFNγ^+^ ex-Th17 cells are associated with different response outcomes to anti-TNF therapy suggests we are dealing with separate groups of patients with subtle but key differences in their disease course. The variable contribution from IL-23 appears to be at least one distinguishing feature that dictates response to anti-TNF therapy.

This study has a number of limitations. While cytokines like TNF-α, IL-1β and IL-6 were prevalent, others were only detected in a small number of participants at baseline, which may reduce statistical power to identify associations. We have made no adjustment for multiple testing of the range of cytokines and cells; therefore, it is possible that these results represent a chance finding and type I error. However, there is biological plausibility to the observed associations, which suggests this is unlikely. There was a wide range of disease duration in our cohort, although two-thirds of participants had disease duration <10 years. This reflects the situation in New Zealand where access to TNF inhibitors is restricted. The cytokine profile in RA may vary over time, and with prior use of DMARDs. For example, methotrexate and leflunomide have been reported to inhibit Janus kinase/signal transducer and activator of transcription (JAK/STAT) signalling and alter TNF and IL-17 production [39, 40]. In addition, the effect from bDMARDs is known to extend to an influence on the transcriptome of both monocytes and CD4^+^ T cells [41]. Finally, we do not have a replication cohort and it is important that these findings are replicated in other populations.

In conclusion, we demonstrate that the presence of plasma IL-23 and circulating IL-17A^+^IFNγ^+^ ex-Th17 cells have the potential to predict disparate outcomes for anti-TNF therapy in RA. IL-23 presence predicts poor outcome, suggesting involvement in a TNF-independent inflammatory mechanism. In contrast, a higher frequency of circulating IL-17A^+^IFNγ^+^ ex-Th17 cells predicts a good outcome from anti-TNF therapy highlighting these cells as a significant component of TNF-mediated inflammation in RA. In combination, plasma IL-23 and circulating IL-17A^+^IFNγ^+^ ex-Th17 cells provide additive value to the prediction of good response to anti-TNF therapy. Further work is required to understand the connection between these two biomarkers, the disease course in RA and the susceptibility to TNF inhibition. Knowledge of joint synovial inflammation associated with these circulating biomarkers may hold the key to understanding this conundrum.

## Conclusions

This study showed that plasma IL-23 and circulating IL-17A^+^IFNγ^+^ ex-Th17 cells are independently associated with response to anti-TNF therapy. Our results suggest the involvement of IL-23 in a TNF-independent inflammatory mechanism in RA, while highlighting IL-17A^+^IFNγ^+^ ex-Th17 cells as a significant component of rheumatoid inflammation that is reliant on TNFa. In combination, plasma IL-23 and circulating IL-17A^+^IFNγ^+^ ex-Th17 cells provide additive value to the prediction of response to anti-TNF therapy in RA.

## Supplementary Information

**Additional file 1.** Antibodies for T-cell identification.

**Additional file 2: Figure S1.** Flow cytometry immunophenotyping strategy.

**Additional file 3.** Detection or concentration of plasma Th17-related cytokines at baseline and EULAR response to anti-TNF therapy.

**Additional file 4.** PBMC cell populations determined by flow cytometry.

## Data Availability

Additional raw datasets collected and/or analysed during the current study are available from the corresponding author on reasonable request. Some restrictions may apply pursuit to ongoing analysis.

## Abbreviations

ACPA: Anti-citrullinated peptide antibody
ACR: American College of Rheumatology
AUC: Area under the curve
bDMARDS: Biological disease-modifying anti-rheumatic drugs
BD: Becton Dickinson
CD: Cluster of differentiation
CRP: C-reactive protein
CI: Confidence interval
DAS28: Disease activity score-4V-CRP
EDTA: Ethylenediaminetetraacetic acid
ELN: Ectopic lymphoid neogenesis
EULAR: European League Against Rheumatism
FCS: Foetal calf serum
FMO: Fluorescence minus one
IFN: Interferon
IL: Interleukin
JAK: Janus kinase
LLOQ: Lower limit of quantitation
MFI: Median fluorescence intensity
OR: Odds ratio
PBMC: Peripheral blood mononuclear cell
PBS: Phosphate-buffered saline
PDPN: Podoplanin
PMA: Phorbol myristate acetate
RA: Rheumatoid arthritis
ROC: Receiver operating characteristic
RORgT: Retinoic acid receptor-related orphan gamma T
sCD40L: Soluble CD40 ligand
STAT: Signal transducer and activator of transcription
Th: T-helper
Treg: T-regulatory
TNF: Tumour necrosis factor

## References

[1] Wijbrandts CA, Tak PP (2017). Prediction of response to targeted treatment in rheumatoid arthritis. Mayo Clin Proc.

[2] van der Pouw Kraan TC (2003). Rheumatoid arthritis is a heterogeneous disease: evidence for differences in the activation of the STAT-1 pathway between rheumatoid tissues. Arthritis Rheum.

[3] Ulfgren AK (2000). Interindividual and intra-articular variation of proinflammatory cytokines in patients with rheumatoid arthritis: potential implications for treatment. Ann Rheum Dis.

[4] Dennis G (2014). Synovial phenotypes in rheumatoid arthritis correlate with response to biologic therapeutics. Arthritis Res Ther.

[5] Orange DE (2018). Identification of three rheumatoid arthritis disease subtypes by machine learning integration of synovial histologic features and RNA sequencing Data. Arthritis Rheumatol.

[6] McKelvey KJ (2018). Co-expression of CD21L and IL17A defines a subset of rheumatoid synovia, characterised by large lymphoid aggregates and high inflammation. PLoS One.

[7] Moelants EA, Mortier A, Van Damme J, Proost P (2013). Regulation of TNF-alpha with a focus on rheumatoid arthritis. Immunol Cell Biol.

[8] Moran EM (2009). Human rheumatoid arthritis tissue production of IL-17A drives matrix and cartilage degradation: synergy with tumour necrosis factor-alpha, Oncostatin M and response to biologic therapies. Arthritis Res Ther.

[9] Lundy SK, Sarkar S, Tesmer LA, Fox DA (2007). Cells of the synovium in rheumatoid arthritis. T lymphocytes. Arthritis Res Ther.

[10] Schett G, Stach C, Zwerina J, Voll R, Manger B (2008). How antirheumatic drugs protect joints from damage in rheumatoid arthritis. Arthritis Rheum.

[11] Wijbrandts CA (2008). The clinical response to infliximab in rheumatoid arthritis is in part dependent on pretreatment tumour necrosis factor alpha expression in the synovium. Ann Rheum Dis.

[12] Lee GR. The balance of Th17 versus Treg cells in autoimmunity. Int J Mol Sci. 2018;19. 10.3390/ijms19030730. 10.3390/ijms19030730 PMC5877591 29510522

[13] Bayry J, Siberil S, Triebel F, Tough DF, Kaveri SV (2007). Rescuing CD4+CD25+ regulatory T-cell functions in rheumatoid arthritis by cytokine-targeted monoclonal antibody therapy. Drug Discov Today.

[14] Kotake S (2016). Elevated ratio of Th17 cell-derived Th1 cells (CD161(+)Th1 cells) to CD161(+)Th17 cells in peripheral blood of early-onset rheumatoid arthritis patients. Biomed Res Int.

[15] Zheng Y (2014). TNFalpha promotes Th17 cell differentiation through IL-6 and IL-1beta produced by monocytes in rheumatoid arthritis. J Immunol Res.

[16] Mazzoni A, Maggi L, Liotta F, Cosmi L, Annunziato F (2019). Biological and clinical significance of T helper 17 cell plasticity. Immunology.

[17] Maggi L (2014). Brief report: etanercept inhibits the tumor necrosis factor alpha-driven shift of Th17 lymphocytes toward a nonclassic Th1 phenotype in juvenile idiopathic arthritis. Arthritis Rheumatol.

[18] Chen DY (2011). Increasing levels of circulating Th17 cells and interleukin-17 in rheumatoid arthritis patients with an inadequate response to anti-TNF-alpha therapy. Arthritis Res Ther.

[19] Alzabin S (2012). Incomplete response of inflammatory arthritis to TNFalpha blockade is associated with the Th17 pathway. Ann Rheum Dis.

[20] Yue C (2010). The effects of adalimumab and methotrexate treatment on peripheral Th17 cells and IL-17/IL-6 secretion in rheumatoid arthritis patients. Rheumatol Int.

[21] Mulhearn B, Barton A, Viatte S. Using the immunophenotype to predict response to biologic drugs in rheumatoid arthritis. J Pers Med. 2019;9. 10.3390/jpm9040046. 10.3390/jpm9040046 PMC6963853 31581724

[22] Aletaha D (2010). 2010 Rheumatoid arthritis classification criteria: an American College of Rheumatology/European League Against Rheumatism collaborative initiative. Arthritis Rheum.

[23] Fransen J, van Riel PL (2005). The Disease Activity Score and the EULAR response criteria. Clin Exp Rheumatol.

[24] Millier MJ, Lazaro K, Stamp LK, Hessian PA. The contribution from interleukin-27 towards rheumatoid inflammation: insights from gene expression. Genes Immun. 2020. 10.1038/s41435-020-0102-z. 10.1038/s41435-020-0102-z 32518420

[25] Bystrom J (2017). Response to treatment with TNFalpha inhibitors in rheumatoid arthritis is associated with high levels of GM-CSF and GM-CSF(+) T lymphocytes. Clin Rev Allergy Immunol.

[26] Shi R, Chen M, Litifu B (2018). Serum interleukin-6 and survivin levels predict clinical response to etanercept treatment in patients with established rheumatoid arthritis. Mod Rheumatol.

[27] Andersson KM (2015). Pathogenic transdifferentiation of Th17 cells contribute to perpetuation of rheumatoid arthritis during anti-TNF treatment. Mol Med.

[28] Hu D (2020). Aberrant expression of USF2 in refractory rheumatoid arthritis and its regulation of proinflammatory cytokines in Th17 cells. Proc Natl Acad Sci U S A.

[29] Basdeo SA (2017). Ex-Th17 (nonclassical Th1) cells are functionally distinct from classical Th1 and Th17 cells and are not constrained by regulatory T cells. J Immunol.

[30] Kotake S, Yago T, Kobashigawa T, Nanke Y. The plasticity of Th17 cells in the pathogenesis of rheumatoid arthritis. J Clin Med. 2017;6. 10.3390/jcm6070067. 10.3390/jcm6070067 PMC5532575 28698517

[31] OIiver J, et al. Transcriptome-wide study of TNF-inhibitor therapy in rheumatoid arthritis reveals early signature of successful treatment. Arthritis Res Ther. 2021;23. 10.1186/s13075-021-02451-9. 10.1186/s13075-021-02451-9 PMC7948368 33691749

[32] Leipe J, Pirronello F, Klose A, Schulze-Koops H, Skapenko A (2020). Increased plasticity of non-classic Th1 cells toward the Th17 phenotype. Mod Rheumatol.

[33] Stamp LK, Easson A, Pettersson L, Highton J, Hessian PA (2009). Monocyte derived interleukin (IL)-23 is an important determinant of synovial IL-17A expression in rheumatoid arthritis. J Rheumatol.

[34] Liu FL (2007). Interleukin (IL)-23 p19 expression induced by IL-1beta in human fibroblast-like synoviocytes with rheumatoid arthritis via active nuclear factor-kappaB and AP-1 dependent pathway. Rheumatology (Oxford).

[35] Canete JD (2015). Ectopic lymphoid neogenesis is strongly associated with activation of the IL-23 pathway in rheumatoid synovitis. Arthritis Res Ther.

[36] Joosten LA (2008). T cell dependence of chronic destructive murine arthritis induced by repeated local activation of Toll-like receptor-driven pathways: crucial role of both interleukin-1beta and interleukin-17. Arthritis Rheum.

[37] Koenders MI (2005). Blocking of interleukin-17 during reactivation of experimental arthritis prevents joint inflammation and bone erosion by decreasing RANKL and interleukin-1. Am J Pathol.

[38] Coutant F, Miossec P (2020). Evolving concepts of the pathogenesis of rheumatoid arthritis with focus on the early and late stages. Curr Opin Rheumatol.

[39] Gonzalez-Alvaro I (2009). Inhibition of tumour necrosis factor and IL-17 production by leflunomide involves the JAK/STAT pathway. Ann Rheum Dis.

[40] Thomas S (2015). Effect of methotrexate on JAK/STAT pathway activation in myeloproliferative neoplasms. Lancet.

[41] Tao W, et al. Multi-omics and machine learning accurately predicts clinical response to adalimumab and etanercept therapy in patients with rheumatoid arthritis. Arthritis Rheumatol. 2020. 10.1002/art.41516. 10.1002/art.41516 PMC7898388 32909363

