# Supplementary material for: Plasma interleukin-23 and circulating IL-17A+IFNγ+ ex-Th17 cells predict opposing outcomes of anti-TNF therapy in rheumatoid arthritis
Source: Arthritis Res Ther. 2022 Feb 26;24:57. doi: 10.1186/s13075-022-02748-3 (PMC8881822; doi:10.1186/s13075-022-02748-3)
Supplement: Supplementary file 1 — Additional file 1. Antibodies for T-cell identification. [file 13075_2022_2748_MOESM1_ESM.docx]

**Additional File 1**

**Antibodies for T-cell identification**

Effector T-cell identification*

| Antibody – fluorochrome conjugate | Clone | Manufacturer |
| --- | --- | --- |
| CD3 – BV510 | UCHT1 | BD Biosciences |
| CD4 – PE-Cy7 | SK3 | BD Biosciences |
| CXCR3 – BV421 | 1C6/CXCR3 | BD Biosciences |
| CD161 – BB700 | DX12 | BD Biosciences (BD Optibuild) |
| CCR4 (CD194) – PE-CF594 | 1G1 | BD Biosciences |
| CCR6 (CD196) – BV650 | 11A9 | BD Biosciences |
| PDPN – AF647 | PDPN/1433 | Novus Biologicals |
| IL-17A – BV785 | BL168 | BioLegend |
| IFNγ – AF488 | #25723 | R&D Systems |
| RORγT – AF700 | #1181A | R&D Systems |

T_reg_ cell identification*

| Antibody – fluorochrome conjugate | Clone | Manufacturer |
| --- | --- | --- |
| CD3 – BV510 | UCHT1 | BD Biosciences |
| CD4 – PE-Cy7 | SK3 | BD Biosciences |
| CD127 – BB700 | HIL-7R-M21 | BD Biosciences |
| CD25 – BV421 | M-A251 | BD Biosciences |
| IL-17A – BV785 | BL168 | BioLegend |
| RORγT – AF700 | #1181A | R&D Systems |
| FOXP3 – AF488 | 236A/E7 | BD Biosciences |

* Fixable viability stain 575V (BD Biosciences) was included within antibody panels
