# Supplementary material for: Plasma interleukin-23 and circulating IL-17A+IFNγ+ ex-Th17 cells predict opposing outcomes of anti-TNF therapy in rheumatoid arthritis
Source: Arthritis Res Ther. 2022 Feb 26;24:57. doi: 10.1186/s13075-022-02748-3 (PMC8881822; doi:10.1186/s13075-022-02748-3)
Supplement: Supplementary file 2 — Additional file 2: Figure S1. Flow cytometry immunophenotyping strategy. [file 13075_2022_2748_MOESM2_ESM.docx]

**
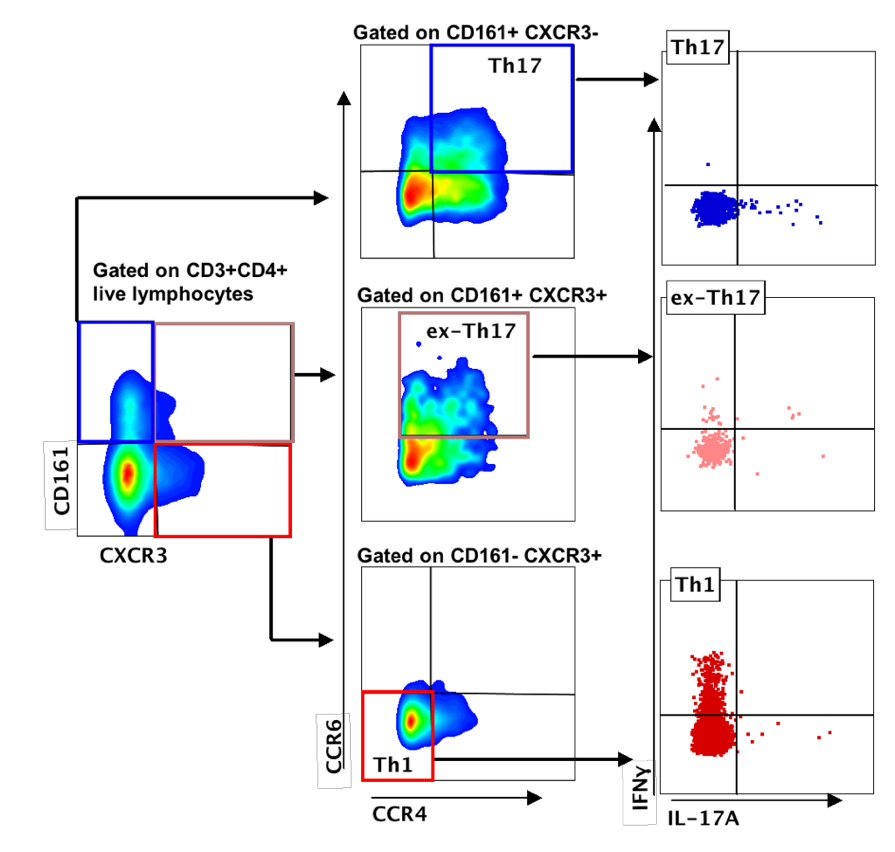
Additional File 2**

**B**

**A**


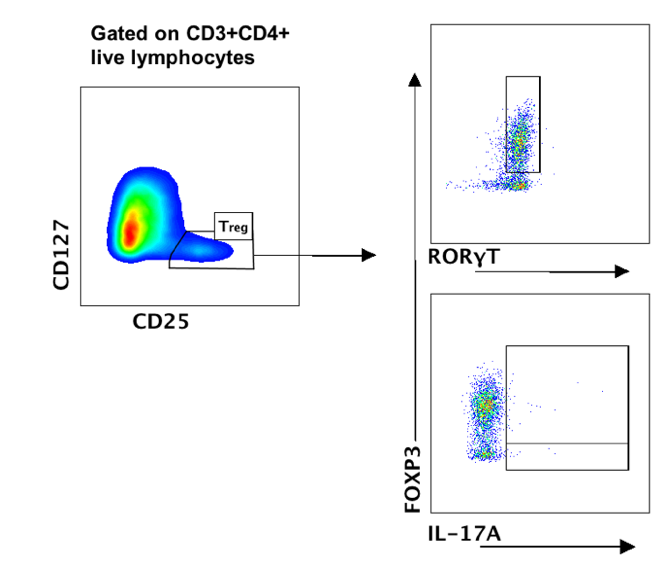


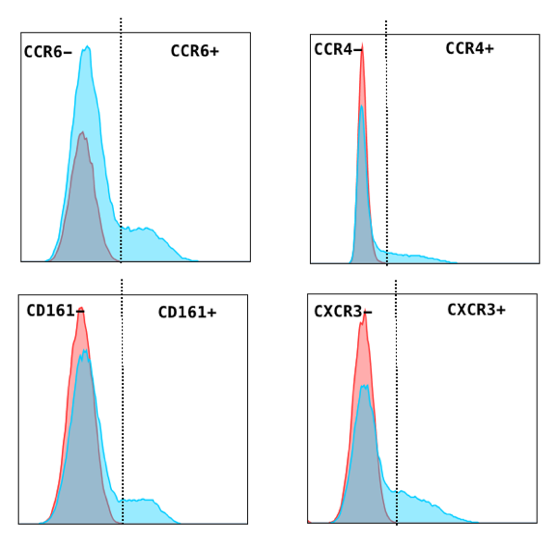


**D**

**C**


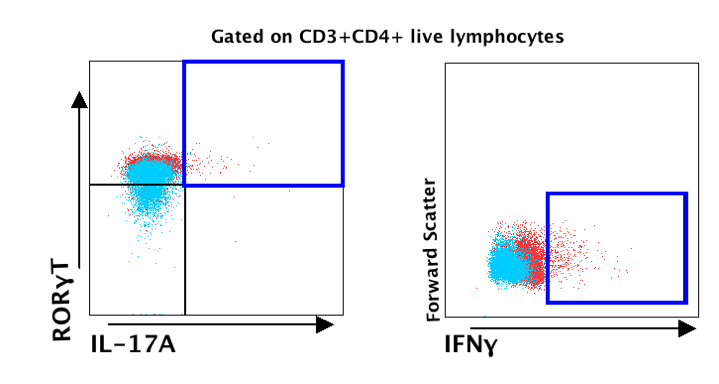


**Supplementary Figure 1. Flow cytometry immunophenotyping strategy.** Multiplex flow cytometry was used to selectively identify the effector T-cells: Th1, Th17, ex-Th17 and regulatory T cells (T_reg_) from within single-event/live/lymphocyte/CD3^+^/CD4^+^ cells based on cell surface marker profile. PBMC samples were stimulated for 6 hr with PMA/ionomycin in the presence of Brefeldin-A, and parallel samples were used for the effector T cell panel (A) and the T_reg_ panel (B).

**A.** Gating strategy for surface marker-defined effector T-cell populations: Th1, CXCR3^+^/CD161^-^/CCR4^-^/CCR6^-^; Th17, CXCR3^-^/CD161^+^/CCR4^+^/CCR6^+^; ex-Th17, CXCR3^+^/CD161^+^/CCR6^+^/CCR4^+/-^. IFNγ and IL-17A were assessed within each effector T-cell population. **B.** Gating strategy for T_reg_ cells: as CD127^lo^/CD25^hi^ with various sub-populations identified based on expression of RORγT, FOXP3 and IL-17A. **C.** Gating thresholds for surface markers. Fluorescence-minus-one (FMO) controls were used to assess staining and establish gating thresholds for surface markers. FMO preparations contained the complete panel of antibodies minus the antibody of interest (red-shaded histogram). Histograms for the sample where the complete marker panel was used are overlaid (blue) and the vertical dashed line demarcates positive from negative populations. **D.** Gating thresholds for intra-cellular cytokine staining. Thresholds were set, determining the positivity of IL-17A and IFNγ specific antibodies, by comparing stimulated and stained samples (red) to unstimulated and stained controls (blue). Additional validation of IL-17A antibody specificity was gained by verifying co-expression of RORγT.
