# Supplementary material for: Plasma interleukin-23 and circulating IL-17A+IFNγ+ ex-Th17 cells predict opposing outcomes of anti-TNF therapy in rheumatoid arthritis
Source: Arthritis Res Ther. 2022 Feb 26;24:57. doi: 10.1186/s13075-022-02748-3 (PMC8881822; doi:10.1186/s13075-022-02748-3)
Supplement: Supplementary file 3 — Additional file 3. Detection or concentration of plasma Th17-related cytokines at baseline and EULAR response to anti-TNF therapy. [file 13075_2022_2748_MOESM3_ESM.docx]

**Additional File 3**

**Detection or concentration of plasma Th17-related cytokines at baseline and EULAR response to anti-TNF therapy**.

|  | **Good Response** | **Poor Response** |  |
| --- | --- | --- | --- |
| Total study sample, n = 93 | 42 (45.2%) | 51 (54.8%) |  |
| Cytokine | Cytokine presence^a^ [N, (%)] |  | p value |
| IL-4 | 9 (50) | 9 (50) | 0.646 |
| IL-17A | 5 (41.7) | 7 (58.3) | 0.794 |
| IL-22 | 9 (40.9) | 13 (59.1) | 0.646 |
| IL-23 | 5 (25) | 15 (75) | 0.041* |
| IFN-γ | 17 (39.5) | 26 (60.5) | 0.312 |
| IL-25 | 22 (50) | 22 (50) | 0.374 |
| CD40-L | 15 (45.5) | 18 (54.5) | 0.966 |
| IL-17F | 12 (42.9) | 16 (57.1) | 0.769 |
| IL-21 | 9 (47.4) | 10 (52.6) | 0.828 |
|  |  |  |  |
| Cytokine | Cytokine concentration^b^  [Median, (IQR)] |  | p value |
| IL-1β | 0.2 (0-0.4) | 0.2 (0-0.4) | 0.555 |
| IL-6 | 18.9 (3.7-47) | 10.4 (2.1-48.1) | 0.533 |
| IL-10 | 2.4 (0-9.5) | 2.6 (0-9.1) | 1.000 |
| IL-31 | 6.7 (0-16.3) | 4.7 (0-27.6) | 0.772 |
| IL-33 | 13.3 (5.1-36.2) | 13.4 (5.4-29.4) | 0.969 |
| TNF-α | 2.9 (2-4.3) | 3 (2.3-4.2) | 0.547 |
|  |  |  |  |

Table shows plasma measures for the presence of cytokine (upper section) or of cytokine concentration (lower section) at baseline, in participants with good response (follow up DAS28 ≤3.2 and ΔDAS28 >1.2) or poor response (follow up DAS28 >3.2 and/or ΔDAS28 ≤1.2) to anti-TNF therapy. Cytokine data was dichotomised to show frequency in cases where ≤ 50% of the total samples had detectable cytokine (upper section) and shows: ^a^the number of samples positive for that cytokine (N) and percentage in parenthesis of the total cytokine positive samples.

^b^ Continuous cytokine concentration is shown as median (pg/ml) with interquartile range (IQR in parenthesis.

Abbreviations: IFN, interferon; IL, interleukin; IQR, interquartile range; TNF, tumour necrosis factor. Pearson’s Chi-Square test was used for comparing frequency and Mann-Whitney U test was used for comparing concentration. *p<0.05 deemed statistically significant.
