# Supplementary material for: Plasma interleukin-23 and circulating IL-17A+IFNγ+ ex-Th17 cells predict opposing outcomes of anti-TNF therapy in rheumatoid arthritis
Source: Arthritis Res Ther. 2022 Feb 26;24:57. doi: 10.1186/s13075-022-02748-3 (PMC8881822; doi:10.1186/s13075-022-02748-3)
Supplement: Supplementary file 4 — Additional file 4. PBMC cell populations determined by flow cytometry. [file 13075_2022_2748_MOESM4_ESM.docx]

**Additional File 4**

**PBMC cell populations determined by flow cytometry.**

|  | **Good Responder (n = 39)** | **Poor Responder (n = 45)** | ***p* value** |
| --- | --- | --- | --- |
| **Lymphocytes:** |  |  |  |
| ^a^CD3^+^CD4^+^ | 53.5 (44.8 - 61.6) | 52.6 (43.7 - 61.1) | 0.968 |
|  |  |  |  |
| **CD3^+^ CD4^+^:** |  |  |  |
| ^a^Th17 | 1.32 (0.73 - 1.94) | 1.37 (0.54 - 2.09) | 0.904 |
| ex-Th17 | 0.64 (0.32 - 1.16) | 0.48 (0.22 - 1.26) | 0.451 |
| Th1 | 8.36 (4.87 – 13) | 9.34 (5.83 - 13.70) | 0.667 |
| T_reg_ | 4.49 (3.7 - 5.44) | 4.46 (3.42 - 5.22) | 0.647 |
| CCR6^+^ | 6.1 (4.2 - 9.19) | 5.91 (3.44 - 9.82) | 0.925 |
| ^b^CCR6^+^ MFI | 1217 (1088 – 1391) | 1141 (968 – 1420) | 0.557 |
| IFNγ+ | 2.11 (1.39 - 3.57) | 2.38 (1.75 - 4.43) | 0.351 |
| IFNγ^+^ MFI | 2523 (2099 – 3256) | 2650 (1976 – 3043) | 0.757 |
| IL-17A^+^ | 0.51 (0.34 - 0.72) | 0.47 (0.32 - 0.85) | 0.833 |
| IL-17A^+^ MFI | 1104 (912 – 1376) | 1076 (974 – 1432) | 0.548 |
| PDPN^+^ | 0.21 (0.12 - 0.82) | 0.21 (0.09 - 0.68) | 0.412 |
| PDPN^+^ MFI | 1658 (994 – 16031) | 1860 (1086 – 17498) | 0.578 |
|  |  |  |  |
| **Th17:** |  |  |  |
| ^a^IFNg^+^ | 0.67 (0.31 - 1.51) | 0.82 (0.43 - 1.37) | 0.706 |
| IFNγ^+^ MFI | 1498 (1214 – 1778) | 1467 (1232 - 1794) | 0.899 |
| IL-17A^+^ | 2.27 (1.14 - 3.46) | 1.95 (1.27 - 2.86) | 0.615 |
| ^b^IL-17A^+^ MFI | 1242 (1038 – 1038) | 1230 (1057 – 1413) | 0.761 |
| IL-17A^+^IFNγ^+^ | 0 (0 - 0.21) | 0 (0 - 0.08) | 0.433 |
| PDPN^+^ | 0.57 (0.22 - 0.92) | 0.62 (0.26 - 1.07) | 0.382 |
| PDPN^+^ MFI | 1498 (942 – 10882) | 1381 (901 – 15857) | 0.907 |
|  |  |  |  |
| **ex-Th17:** |  |  |  |
| ^a^IFNγ^+^ | 8.73 (6.06 - 12.2) | 11 (7.14 - 14.9) | 0.131 |
| ^b^IFNγ^+^ MFI | 1971 (1782 – 2541) | 2100 (1760 – 2797) | 0.586 |
| IL-17A^+^IFNγ^+^ | 0.83 (0.19 - 1.6) | 0.24 (0 - 1.16) | 0.023* |
| IFNγ^+^IL-17A^-^ | 7.08 (5.15 - 10.41) | 9.18 (6.23 - 14.28) | 0.115 |
| IL-17A^+^ | 2.39 (0.95 - 3.93) | 1.82 (0.94 - 4.51) | 0.771 |
| IL-17A^+^ MFI | 1853 (1150 – 3739) | 1567 (1026 – 2068) | 0.244 |
| PDPN^+^ | 0.67 (0 - 1.72) | 0.5 (0 - 1.7) | 0.953 |
| PDPN^+^ MFI | 1339 (882 - 8798) | 1171.5 (883 - 3808) | 0.906 |
|  |  |  |  |
| **Th1:** |  |  |  |
| ^a^IFNγ^+^ | 9.42 (6.33 – 19.0) | 10.7 (8.61 - 14.3) | 0.479 |
| ^b^IFNγ^+^ MFI | 2670 (2048 – 3128) | 2419 (2017 – 2890) | 0.654 |
| IL-17A^+^IFNγ^+^ | 0.088 (0.02 - 0.14) | 0.067 (0.014 - 0.13) | 0.517 |
| IL-17A^+^ | 0.6 (0.36 - 0.97) | 0.73 (0.27 - 1.23) | 0.737 |
| IL-17A^+^ MFI | 1028 (817 – 1418) | 1078 (877 – 1652) | 0.240 |
| PDPN^+^ | 0.6 (0.23 - 1.49) | 0.36 (0.2 - 1.17) | 0.282 |
| PDPN^+^ MFI | 1459 (730 – 13061) | 1680 (798 - 12532) | 0.752 |
|  |  |  |  |
| **T_reg_:** |  |  |  |
| RORγT^+^ FOXP3^+^ | 70.05 (61.3 - 75.1) | 67.2 (61.1 - 74.3) | 0.814 |
| FOXP3^+^IL-17A^+^ | 0.64 (0.27 - 0.95) | 0.5 (0.31 - 0.84) | 0.504 |
| ^c^FOXP3^+^IL-17A^+^ MFI | 958 (758 – 1290) | 1089 (840 – 1246) | 0.373 |
| FOXP3^-^ IL-17A^+^ | 0.23 (0.11 - 0.38) | 0.23 (0.08 - 0.38) | 0.726 |
| ^c^FOXP3^-^ IL-17A^+^ MFI | 1283 (873 – 1715) | 1218 (958 – 2098) | 0.521 |
|  |  |  |  |
| **Cell Ratios:** |  |  |  |
| Th1: Th17 | 6.0 (4.4 - 12.8) | 7.3 (3.7 - 18.9) | 0.69 |
| Th1: ex-Th17 | 12.1 (7.3 - 24.5) | 16.9 (9.1 - 32.1) | 0.422 |
| Th17: ex-Th17 | 1.8 (1.4 - 4.7) | 1.9 (1.1 - 5.2) | 0.854 |
| T_reg_: Th17 | 3.7 (2.5 - 5.3) | 4.1 (2.1 – 6.0) | 0.897 |

**^a^** Values for indicated cellular PBMC sub-populations are median (IQR) frequencies (%) of the indicated parent cell populations (shown in bold). ^b^ Median fluorescence intensity (MFI) is the median MFI value of indicated positive cell sub-populations. ^c^ MFI of IL-17A expression only in double positive cells.
